# Assessment of Obesity by Using Various Anthropometric Measurements among Patients with Coronary Heart Disease Residing in North India

**DOI:** 10.7759/cureus.7948

**Published:** 2020-05-03

**Authors:** Nisha Golia, Kewal Krishan, Jeet Ram Kashyap

**Affiliations:** 1 Anthropology, Panjab University, Chandigarh, IND; 2 Cardiology, Government Medical College and Hospital Sector, Chandigarh, IND

**Keywords:** obesity, coronary heart disease, body mass index, waist circumference, waist hip ratio, body fat percentage, visceral fat percentage, anthropometric measurements

## Abstract

Background

Obesity is one of the main risk factors of coronary heart disease (CHD). Although a range of anthropometric measures are available to evaluate obesity, which measure is the most precise to predict the risk of CHD is still controversial. Therefore, we assess the prevalence of obesity among patients with CHD by using various anthropometric techniques to find out the most efficient method to predict the risk of CHD.

Methods

In this cross-sectional study, we included 300 CHD patients and 100 age and sex-matched healthy controls, aged 45-70 years. Various anthropometric measurements (waist and hip circumference, waist-hip ratio, body mass index, and body fat percentage) were taken to assess the prevalence of obesity among the selected population.

Results

Average waist circumference among male and female patients was significantly higher than the controls; 94.0±13.2 vs. 86.4±4.4 (*p *< 0.001) and 97.8±12.1 vs. 86.9±5.3 (*p *< 0.001) respectively. The average waist-hip ratio among patients of both genders was significantly higher than controls 1.0±.06 vs. 0.92±.04 (*p *< 0.001) among males and 0.96±.07 vs. 0.88±.04 (*p *< 0.001) among females, respectively. The average body mass index (BMI) was not different among male patients (24.6±4.0) compared to controls (24.3±2.3); however, the frequency distribution of BMI among male patients and controls was significantly different (*p *< 0.05), whereas female patients had significantly higher BMI compared to controls 27.7±4.9 and 25.1±2.4, (*p *< 0.001). Similarly, body fat percentage and visceral fat percentage were elevated among female cases vs. female controls, but no significant difference was observed in the body fat percentage of male cases vs. controls 28.0±5.0 vs. 28.1±2.7; (*p *> 0.05). However, visceral fat percentage was significantly elevated among male cases vs. controls 11.6±5.7 vs. 9.6±2.6 (*p *< 0.05).

Conclusions

Central adiposity markers, waist circumference (WC), waist-hip ratio (WHR), and visceral fat percentage were uniformly present in patients of both sexes and are stronger predictors of risk of CHD relative to the BMI.

## Introduction

Coronary heart disease (CHD) is one form of cardiac disease. The pathogenesis is attributed to atherosclerosis, resulting in the formation of plaques which further lead to a compromise in the availability of oxygenated blood to the cardiac muscles [1]. Atherosclerosis starts as a minimum inflammatory condition of the inner lining of the endothelial wall that proliferates when subjected to various risk factors such as obesity, hypertension, smoking, dyslipidemia, diabetes, and other provocative factors [2-3]. CHD is a major cause of mortality around the globe among non-communicable diseases. The mortality rate due to CHD rose from 14.6 percent to 18.6 percent between 2005 and 2015 causing an estimated nine million deaths globally [4]. In India, the burden of CHD has also increased to double the mortality rate documented in 1990, owing to rapid urbanization and lifestyle alterations. In 2017, CHD accounted for 15.5 percent of overall mortality rate which is almost identical to the global burden of CHD i.e. 15.9 percent, accounting 17.5 percent of deaths in males and 13.3 percent of deaths in females in India [5-6].

Obesity is a major risk factor for cardiovascular disease (CVD) including CHD, heart failure (HF), hypertension (HT), and arrhythmias. Increased body weight is also associated with increased coronary events and mortality among heart disease patients [7-8]. Globally, elevated body mass index (BMI) accounted for 148 million disability-adjusted life years (DALYs) along with 4.7 million deaths, increased 70% since 1990, and was the 4th highest cause of death, in 2017 [9]. The National Family Health Survey-4 (NFHS) reported 20.6% of all women as overweight, accounting for 31.3% urban and 15.0% rural women, whereas 18.9% of men were found to be overweight, accounting for 26.6% urban and 14.3% rural men in India [10]. Elevated BMI is a known risk factor associated with HT, dyslipidemia, and hyperglycemia as well as increased risk of CHD [11]. Central obesity measures including waist circumference (WC) and waist-hip ratio (WHR) are considered better markers of the risk of CHD than BMI among obese people [12]. WHR is an easy, practical, and low-cost technique to determine visceral fat (VF) [13]. Elevated WHR raises the risk of CVD fatality and disability via changing the autonomic function in the heart and is a stronger predictor of visceral obesity and poor cardiac health than BMI [14]. Cardio-metabolic problems associated with adiposity are determined by fat distribution rather than total body fat [15]. This is attributed to an imbalance in the production of inflammatory and anti-inflammatory adipokines [16]. Anthropometric measurements are among the simplest and low-cost methods to measure obesity and the risk of CHD among mass populations. There has always been a disagreement among researchers about which anthropometric measurements are more precise and accurate to predict the risk of CHD. Therefore, the present study attempts to assess the accuracy of predicting the prevalence of obesity among CHD patients by using various anthropometric measurements.

## Materials and methods

A cross-sectional study was carried out on newly diagnosed CHD patients based upon symptoms, finding of non-invasive tests, and angiographic evidence of CAD residing in the northwest region of India. Data were collected from 300 symptomatic CHD patients who visited the Cardiology OPD and Cardiac Care Unit of Government Medical College and Hospital, Chandigarh, India. Age and gender-matched asymptomatic subjects with no clinical evidence of CHD were recruited as controls (n-100). Patients with other co-morbidities including fever, infections, autoimmune disorders, and respiratory and gastrointestinal distress were excluded. Ethical clearance for collecting data from human subjects was obtained from the Institutional Ethics Committee (IEC), Panjab University, Chandigarh. Furthermore, ethical approval from the IEC of GMCH was also obtained to conduct fieldwork. The patient consent form was read to the participants in vernacular language (Hindi, English, and Punjabi) and their duly signed consent was taken before participation. It was clearly explained to the subjects that their identity will be kept confidential.

Selected subjects were interviewed to obtain primary data for the study. Anthropometric measurements such as BMI, WC, WHR, and bioelectrical impedance analysis (BIA) were collected. Bodyweight (kg) was recorded with the help of a portable electric scale (Omron H-301 Korea). Total standing height was measured from the top of the head (Vertex) till foot-sole, using a portable anthropometric rod (GPM type Anthropometer, Galaxy Informatics, New Delhi) [17]. The waist circumference (WC, in cm) was measured with a stretch proof tape, at the midpoint between the costal margin and iliac crest, and the hip circumference (HC, in cm) was measured at the widest point of the buttocks by placing the measuring tape parallel to the floor, as per the World Health Organisation (WHO) guidelines [18-19]. The WC was divided by HC to calculate the waist-hip ratio (WHR). The body mass index (BMI) was calculated by dividing body weight (kg) with the square of the height (m^2^) and classification for Asians was used in the study [20]. The body fat percentage and visceral fat percentage were measured using a bioelectric impedance analyzer (Omron HBF-701). The statistical analysis was done by using the statistical program for Social Sciences (SPSS) version 18 and descriptive stats were executed to examine the general tendencies of data groups. An Independent t-test was applied to compare the mean values of various anthropometric measurements of cases and controls. Besides, Pearson’s chi-square test (*X*^2^) was applied to observe the frequency distribution of BMI among cases and controls.

## Results

As shown in Table 1, a total of 300 patients presenting with CHD were included in the study comprising 169 males (56.3%) and 131 females (43.6%), aged between 45 and 70 years. The average age was recorded to be 56 years in males and 57 years among females. Among these, 57 male patients (33.7%) and 35 female patients (26.7%) were hypertensive, and 58 male patients (34.3%) and 62 female patients (47.3%) had diabetes mellitus. Additional baseline characteristics of patients and controls who participated in the study are shown in Table 1.

**Table 1:** Baseline characteristics of participants N – number; Values in parentheses are percentages; Other values are mean ± standard deviation, (*p *< 0.05)

Characteristics	Male patients N=169 (56.3%)	Male controls N=50 (50%)	p-value	Female patients N=131 (43.6%)	Female controls N=50 (50%)	p-value
Age (years)	56.5±8.3	56.2±6.3	0.821	57.4±7.7	57.4±7.1	0.975
Hypertension	57 (33.7)	2 (4)	0.000	35 (26.7)	0	0.000
Diabetes mellitus	58 (34.3)	0	0.000	62 (47.3)	0	0.000
Cholesterol (mg/dl)	157.1±41.0	146.1±7.4	0.002	166.5±49.3	150.5±18.5	0.039
Triglycerides (mg/dl)	161.7±79.8	107.4±9.4	0.000	140.3±58.9	114.9±14.9	0.024
Low-density lipoproteins (mg/dl)	91.8±36.5	74.7±18.0	0.003	99.0±42.8	79.1±24.9	0.019
High-density lipoproteins (mg/dl)	44.0±14.0	60.0±3.6	0.000	48.6±15.3	61.5±7.0	0.000
Systolic blood pressure (mm/Hg)	135.6±22.4	114.3±6.8	0.000	137.1±21.0	118.0±8.2	0.000
Diastolic blood pressure (mm/Hg)	83.5±11.4	77.8±4.6	0.000	81.3±11.3	77.5±4.0	0.002
Fasting blood sugar (mg/dl)	144.5±55.6	97.4±12.8	0.000	162.8±63.1	108.5±14.6	0.000

A comparison between CHD patients and controls regarding the frequency distribution of BMI according to Asian classification is shown in Table 2 [20]. According to the results, 5 (3%) male and 1 (0.8%) female patients were underweight, however, no one was found underweight in control groups. In the case of males, 59 (34.9%) patients vs. 18 (36%) controls had normal BMI, whereas 25 (19.1%) female patients vs. 12 (24%) female controls had BMI within the normal range. In males, 71 (42%) patients vs. 32 (64%) controls were overweight, while 37 (28.2%) female patients vs. 32 (64%) female controls were overweight. Obesity class-1 (BMI: 27.5-32.5 kg/m^2^) was observed in 28 (16.6%) male patients, 45 (34.4%) female patients, and 6 (12%) female controls, whereas obesity class-I was not present among the male control group. According to the results, obesity class-II (BMI: 32.5-37.5 kg/m^2^) was reported among 5 (3.0%) male patients and 19 (14.5%) female patients, similarly, obesity class-III (BMI>37.5+ kg/m^2^) was observed among 1 (0.6%) male patients and 4 (3.1%) female patients but none among control groups. Pearson’s chi-square test (*X*^2^) was applied to the frequency distribution of BMI among cases and controls. The frequency distribution of BMI was significantly different among male cases and controls (*p* < 0.05) and obesity (BMI: 27.5-37.5 kg/m^2^) was prevalent among 34 (20.2%) male patients. In contrast, obesity (BMI: 27.5-37.5 kg/m^2^) was even more prevalent among 68 (52%) female cases in comparison to 6 (12%) female controls, and frequency distribution of BMI was highly significant (*p *< 0.001) among them.

**Table 2:** Frequency distribution of body mass index among CHD patients and controls N: Number; values in parentheses are percentages; **p<0.001; *p<0.05 CHD, coronary heart disease

BMI Status	Male patients N = 169	Male controls N = 50	p-value	Female patients N = 131	Female controls N = 50	p-value
Underweight (<18.5 kg/m^2^)	5 (3.0)	0	<0.05*	1 (0.8)	0	<0.001**
Normal ( 18.5-23 kg/m^2^)	59 (34.9)	18 (36.0)		25 (19.1)	12 (24.0)	
Overweight ( 23-27.5 kg/m^2^)	71 (42.0)	32 (64.0)		37 (28.2)	32 (64.0)	
Obese Class-I ( 27.5-32.5 kg/m^2^)	28 (16.6)	0		45 (34.4)	6 (12.0)	
Obese Class-II ( 32.5-37.5 kg/m^2^)	5 (3.0)	0		19 (14.5)	0	
Obese Class-III ( >37.5^+ ^kg/m^2^)	1 (0.6)	0		4 (3.1)	0	

The descriptive analysis of anthropometric measurements, as well as body composition indices of CHD patients and controls, is presented in Table 3. The average WC of male as well as female patients was significantly higher than controls 94.0±13.2 vs. 86.4±4.4 (*p *< 0.001) and 97.8±12.1 vs. 86.9±5.3 (*p *< 0.001), respectively. Similarly, the average WHR among patients of both genders was significantly higher than controls, 1.0±.06 vs. 0.92±.04; (*p *< 0.001) among males and 0.96±.07 vs. 0.88±.04 (*p *< 0.001) among females, respectively. The average BMI was not different among male patients 24.6±4.0 compared to male controls 24.3±2.3 (*p *> 0.05), whereas, the female patients had a significantly higher average BMI compared to female controls, 27.7±4.9 and 25.1±2.4, respectively (*p *< 0.001). The average BF % was identical among male patients 28.0±5.0 compared to male controls 28.1±2.7 (*p *> 0.05), however, the female patients had significantly higher average BF % compared to female controls, 38.3±5.0 and 32.8±2.3, respectively (*p *< 0.001). Similarly, the average VF % among patients of both genders were significantly higher than controls 11.6±5.7 vs. 9.6±2.6 (*p* < 0.05) among males and 11.9±5.9 vs. 7.9±2.8 (*p *< 0.001) among females, respectively.

**Table 3:** Descriptive analysis of anthropometric measurements and body composition of CHD patients and controls WC - waist circumference; HC - hip circumference; WHR - waist-hip ratio; BMI - body mass index; Body fat% - BF%; CHD - Coronary heart disease; Visceral fat% - VF%; **p < 0.001, *p < 0.05

Characteristics	Male patients N=169	Male controls N=50	p-value	Female patients N=131	Female controls N=50	p-value
Weight (kg)	68.2±12.5	69.2±6.8	.614	65.7±13.1	63.5±7.9	0.282
Height (cm)	166.3±6.5	168.8±7.4	.023	153.7±6.4	158.7±5.5	0.000
WC (cm)	94.0±13.2	86.4±4.4	.000	97.8±12.1	86.9±5.3	0.000
HC (cm)	91.8±11.6	93.8±6.6	.117	102.0±12.7	98.6±4.8	0.040
WHR	1.0±0.06	0.92±.04	.000	0.96±0.07	0.88±.04	0.000
BMI (kg/m^2^)	24.6±4.0	24.3±2.3	.602	27.7±4.9	25.1±2.4	0.001
BF %	28.0±5.0	28.1±2.7	.912	38.3±5.0	32.8±2.3	0.000
VF %	11.6±5.7	9.6±2.6	.018	11.9±5.9	7.9±2.8	0.000

## Discussion

In our study, we attempted to determine the prevalence of obesity by using various anthropometric measurements among CHD patients, to identify the risk of CHD. Our results show that obesity from class I-III (BMI: 27.5-37.5 kg/m^2^) was prevalent only among 34 (20.2%) male patients, however, 135 (79.9%) male patients were below obesity range (BMI <27.5 kg/m^2^) according to Asian BMI classification [20]. In our study, the average BMI was not different among male patients compared to controls, regardless of that, their average WC, WHR, and VF % were significantly higher than male controls. The outcomes indicate the prevalence of central adiposity among male patients even at lower BMI. However, obesity from class I-III (BMI: 27.5-37.5 kg/m^2^) was even more prevalent among 68 (52%) female patients in contrast to 6 (12%) female controls. Furthermore, BMI >30 kg/m^2^ has been reported as an independent risk factor for the occurrence of coronary diseases [21]. Besides, individuals with BMI <17.5 or >24.9 kg/m^2^ have also been linked with increased CVD mortality among the East Asian population [22]. It is therefore evident from previous studies that there is an increased risk of CHD in both under-nourished as well as over-nourished individuals. Also, a considerable correlation has been found between BMI with fasting blood sugar levels (FBS), blood pressure (BP), and lipid profile among females and non-smoker males [23]. Hence, using only BMI to predict the risk of mortality among CHD patients can be ambiguous. Therefore, other procedures of measuring trunk adiposity must be applied along with BMI to predict the long term risk of mortality among CHD patients [24].

Our study found that central obesity was relatively prevalent among CHD patients since their average WC was above reference cut-off criteria for south Asians (male >90 cm; female >80 cm). In addition, CHD patients’ WHR was also reported above WHO reference range (male >0.9; female >0.85) [18, 19]. Moreover, heredity disposition towards central adiposity adjusted to BMI raises the prevalence of CHD [25]. In concurrence with our findings, the Chennai CURES study reported that Asian Indians are highly prone towards mid-waist obesity than the US population at lower BMI; hence, CVDs show higher prevalence among Asian Indians as compared to Caucasians with similar BMI [26].

Based on our study, female patients had significantly higher BF % than female controls however, no major difference was observed in BF % of male patients and male controls. Additionally, raised body fat % has been correlated with an increased probability of a cardiac event, while higher fat-free mass has been correlated with the inferior chance of a cardiac event in CHD patients. Therefore, BF %, not BMI is linked with “major adverse cardiovascular events” [27]. 

In our study, male as well as female patients had significantly higher VF % than controls. Hypertension, ischemic heart disease (IHD), diabetic cardiomyopathy, and elevated VF have been associated with coronary events. The VF stores, including fat build-up in epicardial linings, are linked with higher CVD risk. This results from the effect of increased lipid emission, pro-inflammatory factors, and adipokines from adipose tissues [28]. Interestingly, patients with epicardial fat build-ups have a greater threat of developing atherosclerosis even in the absence of an increase in overall VF [29].

Results of the present study indicate that WC, WHR, and VF % measurements were uniformly elevated among CHD patients than controls in both genders. Whereas, average BMI was similar among male cases and controls and showed no signification difference, conversely, the frequency distribution of BMI was significantly different among male cases and controls, thus the outcomes were found to be ambiguous to apply for CHD risk assessment. People with identical bodyweight may have immeasurable diversity in their body composition as BMI does not differentiate within the lean mass, fat mass, and fat-free mass [30]. Therefore, BMI may not be a true indicator of central obesity and visceral fat distribution (potential risk factors of CHD) among individuals. Although BMI doesn't actually assess body fat, it can qualify as an initial investigating tool of obesity assessment but not for risk evaluation among CHD patients. Since no single anthropometric measurement can accurately distinguish CHD risk; hence, it is suggested to apply central adiposity measures alongside for better assessment of obesity among CHD patients for preventive outcomes relative to solely using BMI. 

Our study has some limitations. First, in this cross-sectional research, we did not have the opportunity to examine the cause and effect association between obesity and CHD. Besides, the sample size of the control group can be taken as a restricting factor. The changed body water distribution among people is one of the constraining factors to utilize BIA in the assessment of body fat and obesity. The results of this research may be inadequate for the diverse cultures, demographic groups, and metropolitan communities in India. The anthropometric measurements could not be evaluated for sensitivity, specificity, positive predictive value, and negative predictive values to examine the precision of these measurements. However, the anthropometric measurements were taken by a trained anthropometrist (NG) by following all the standard procedures and protocols. Therefore, further researches are required in line to explore a greater understanding of the accuracy and precision of these methods.

## Conclusions

Anthropometric measurements are a simple and inexpensive tool for assessing adiposity and risk of CHD. Our results also show that there are wide variations between populations, in the effect of BMI, WC, WHR and, VF% on predicting the risk of CHD. While BMI is a useful tool to assess obesity in the mass population, it should be used in conjunction with other markers of central adiposity (WC, WHR, and VF %) for better prediction of CHD risk. It is therefore important to conduct population-specific studies and develop country-wise or even province-specific references for predicting CHD risk using these anthropometric measurements of predicting central adiposity.
